# Optical and Structural Properties of Aluminum Nitride Epi-Films at Room and High Temperature

**DOI:** 10.3390/ma16237442

**Published:** 2023-11-30

**Authors:** Yanlian Yang, Yao Liu, Lianshan Wang, Shuping Zhang, Haixia Lu, Yi Peng, Wenwang Wei, Jia Yang, Zhe Chuan Feng, Lingyu Wan, Benjamin Klein, Ian T. Ferguson, Wenhong Sun

**Affiliations:** 1 Research Center for Optoelectronics Materials and Devices, Guangxi Key Laboratory for the Relativistic Astrophysics, School of Physical Science and Technology, Guangxi University, Nanning 530004, China; kindy789456@126.com (Y.Y.); ls-wang@semi.ac.cn (L.W.); spz0909@163.com (S.Z.); 18327851334@163.com (H.L.); 1907401031@st.gxu.edu.cn (Y.P.); weiww9189@163.com (W.W.); yangjia1548668228@163.com (J.Y.); 2 Key Laboratory of Semiconductor Materials Science, Institute of Semiconductors, Chinese Academy of Sciences, Beijing 100083, China; 3 College of Materials and Chemical Engineering, Hezhou University, Hezhou 542700, China; 4 Southern Polytechnic College of Engineering and Engineering Technology, Kennesaw State University, Kennesaw, GA 30144, USA; zfeng6@kennesaw.edu (Z.C.F.); bklein8@kennesaw.edu (B.K.); ianf@kennesaw.edu (I.T.F.); 5 Science Exploring Laboratory, Arbour Glenn Drive, Lawrenceville, GA 30043, USA; 6 Center on Nano-Energy Research, Laboratory of Optoelectronic Materials and Detection Technology, Guangxi Key Laboratory for the Relativistic Astrophysics, School of Physical Science and Technology, Guangxi University, Nanning 530004, China; wanlingyu75@126.com; 7 State Key Laboratory of Featured Metal Materials and Life-Cycle Safety for Composite Structures, MOE Key Laboratory of New Processing Technology for Nonferrous Metals and Materials, Guangxi Key Laboratory of Processing for Nonferrous Metals and Featured Materials, Guangxi University, Nanning 530004, China

**Keywords:** aluminum nitride, spectroscopic ellipsometry, temperature-dependent SE, Urbach’s binding energy

## Abstract

The high-quality aluminum nitride (AlN) epilayer is the key factor that directly affects the performance of semiconductor deep-ultraviolet (DUV) photoelectronic devices. In this work, to investigate the influence of thickness on the quality of the AlN epilayer, two AlN-thick epi-film samples were grown on c-plane sapphire substrates. The optical and structural characteristics of AlN films are meticulously examined by using high-resolution X-ray diffraction (HR-XRD), scanning electron microscopy (SEM), a dual-beam ultraviolet-visible spectrophotometer, and spectroscopic ellipsometry (SE). It has been found that the quality of AlN can be controlled by adjusting the AlN film thickness. The phenomenon, in which the thicker AlNn film exhibits lower dislocations than the thinner one, demonstrates that thick AlN epitaxial samples can work as a strain relief layer and, in the meantime, help significantly bend the dislocations and decrease total dislocation density with the thicker epi-film. The Urbach’s binding energy and optical bandgap (*E_g_*) derived by optical transmission (OT) and SE depend on crystallite size, crystalline alignment, and film thickness, which are in good agreement with XRD and SEM results. It is concluded that under the treatment of thickening film, the essence of crystal quality is improved. The bandgap energies of AlN samples obtained from SE possess larger values and higher accuracy than those extracted from OT. The Bose–Einstein relation is used to demonstrate the bandgap variation with temperature, and it is indicated that the thermal stability of bandgap energy can be improved with an increase in film thickness. It is revealed that when the thickness increases to micrometer order, the thickness has little effect on the change of *E_g_* with temperature.

## 1. Introduction

With the rapid development of energy conservation and emission reduction, electric vehicles, rail transit, new energy power generation, smart grid and wireless communication, and other technical fields, industry is increasingly demanding the performance index and reliability of power semiconductor devices [1,2,3,4,5]. Aluminum nitride (AlN) is a kind of ultra-wide bandgap material with a large bandgap (*E_g_*) (6.2 eV), high critical electric field, and high breakdown voltage for a variety of electronic and photonic applications. Since epitaxially grown AlN is best matched to the lattice of compound semiconductors such as binary Gallium nitride (GaN) or terpolymer Gallium aluminum nitride (AlGaN). It is commonly used to prepare ultraviolet (UV) light-emitting diodes (LEDs) and GaN/AlGaN-based electronic and photonic devices based on III-Nitrides. Large numbers of studies have shown that the high fault, strain and stress in the AlN template layer will affect the luminescence and electronic characteristics of the device, and the quality of the AlN template material will significantly affect the device’s performance [6,7]. Therefore, high-quality aluminum nitride is required for better device performance. Previously, different techniques, such as substrate pretreatment, interlayer insertion, annealing of nucleation layers, and strain management, have been reported to improve the quality of AlN films grown on sapphire and silicon carbide (SiC) substrates [8,9,10,11,12,13,14]. In recent years, considerable studies have been performed on AlN by our research team [15,16,17,18,19]. Research and development (R&D) on AlN is currently still at the frontier of scientific society [6,20,21,22,23,24,25,26,27].

Extensive reports have used traditional methods such as high-resolution X-ray diffraction (HR-XRD), optical transmission (OT), and Raman spectroscopy to evaluate the structure and optical characteristics of AlN [15,18,28,29,30]. Samiul Hasan et al. [28] investigated a 4 μm thick crack-free AlN film grown on a 0.2° offcut sapphire substrate using nitrogen as a carrier gas. They analyzed the structural properties of AlN at room temperature using atomic force microscopy (AFM), XRD, and Raman spectroscopy but did not study them at high temperatures. Nam et al. [29] reported the energy bandgap (*E_g_*) with changing temperature in the AlN epilayer from 10 K to 800 K measured by deep ultraviolet (DUV) photoluminescence (PL) spectroscopy. Because the bandgap energy evaluated from the analysis of reflectance spectra and photoluminescence is different, the PL measurement method can accurately measure the temperature-dependent inter-band transition energy associated with *E_g_* [30] but cannot evaluate the Urbach band tail effects below the bandgap edge. Recently, Zhe Chuan Feng et al. [15] reported optical studies of AlN films with different thicknesses (0.4–10 µm) grown on sapphire using XRD, OT, SE, and Raman. However, Urbach’s energy was simply studied by the spectroscopic ellipsometry (SE) method and the variation trend of the energy bandgap with temperature was not studied under high-temperature conditions. Few studies are using XRD, OT, and SE methods simultaneously to characterize the grain crystal quality of AlN thin films. The Urbach’s binding energy (E_u_) obtained by OT and high-temperature SE methods can be used to analyze the intrinsic effects of thin film crystal quality. When studying the optical properties of semiconductor thin films, the high-temperature ellipsometry method can be used to obtain the thickness of AlN thin films more accurately and the changes in refractive index, absorption edge, and bandgap of each layer with temperature.

In the present work, the optical and structural properties of two AlN films grown on sapphire by MOCVD are investigated using HRXRD, OT, scanning electron microscopy (SEM), and SE, especially focused on optical properties at high temperatures by temperature-dependent SE from 300 K to 850 K. These AlN epitaxial layer samples were obtained with low defect density, low residual stress, low strain field, and no cracks.

## 2. Materials and Methods

The thick AlN epi-films were epitaxial on vicinal sapphire (0001) (c-plane) substrates by MOCVD and by using trimethyl-aluminum (TMAl) and ammonia (NH_3_) as Al and N precursors, respectively. The deposition was started by the step-growth method. First, a low-temperature (LT) AlN inter-layer, followed by a high-temperature (HT) AlN epitaxial layer. The two samples are named AlNt and AlNn, with nominal-designed thicknesses of 2 and 4 μm, respectively.

The optical and structural characteristics of AlN epilayers are examined by using HR-XRD, a dual-beam ultraviolet-visible (UV-Vis) spectrophotometer, scanning electron microscopy (SEM), and spectroscopic ellipsometry (SE). The experiment data was analyzed to acquire the optical constants, bandgap, Urbach’s energy, thickness, and surface roughness of AlN films. The crystalline perfection and related characteristics of the AlN films were evaluated by high-resolution X-ray diffraction (HR-XRD, *λ* = 0.15406 nm, X’Pert3 MRD) with a 2*θ* = 0.002° scanning step. The dual-beam UV-Vis spectrophotometer (TU1901) was used to measure optical transmission (OT) spectra in the wavelength region from 190 to 850 nm by placing the sample in a black box. SEM was employed to measure the film cross-section structural information. The SE spectral data were measured from a Mueller matrix ellipsometer (Wuhan Eoptics Technology Co. Ltd., Wuhan, China) at five incidence angles of 60°, 65°, and 70°, with the temperature variation in the range of 300 K to 850 K, controlled by a Linkam temperature stage (THMS600). Temperature-dependent SE measurements of AlN films were performed after stabilizing each temperature point for 15 min to ensure the uniformity and accuracy of the surface temperature.

## 3. Results and Discussion

### 3.1. Structural Characterization by High-Resolution X-ray Diffraction

**Figure 1:**
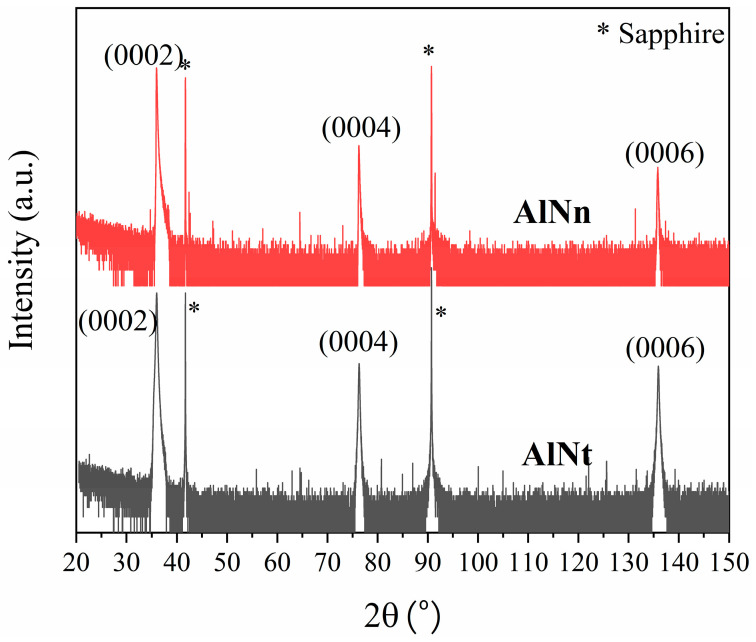
XRD-wide scans of AlNt and AlNn, showing AlN (0002), (0004), and (0006).

High-resolution X-ray diffraction (HR-XRD) is an effective method for understanding the crystal structure and quality. The orientation of the AlN epitaxial layer can be determined by 2*θ*-ω scanning of the sample using HR-XRD. Figure 1 shows the AlN (0002), (0004), and (0006) patterns for two AlN samples, characteristic of their crystalline wurtzite structure. It is determined that the two AlN films on sapphire show an obvious polarity (0001) orientation from the characteristic peaks of AlN and sapphire.

**Figure 2:**
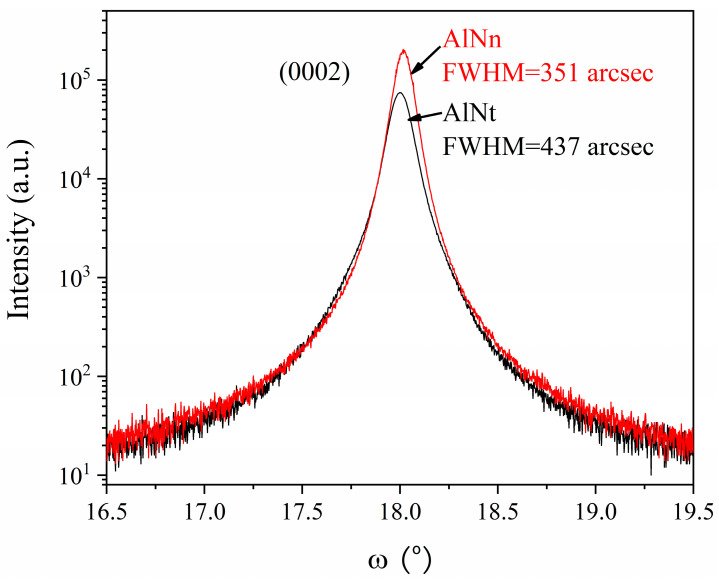
(0002) XRC of AlNt and AlNn by HR-XRD.

To analyze the structural characteristics of two AlN samples and compare their crystal quality, the X-ray rocking curves (XRC) of two samples were tested. As shown in Figure 2, the crystal orientation (0002), (0004), and (0006) rocking curves of samples are measured, and the FWHM values are shown in Table 1, which were obtained by Gaussian fitting.

The average grain size of the AlNt and AlNn can be estimated by the Debye–Sheller formula [16,31]:

(1) $$D=\frac{k\lambda }{\beta cos\theta }$$

where *D* is the grain size (diameter), *β* is the half-peak width of (0002) XRC, *k* is the Scherrer constant (*k =* 0.9), *λ* is the X-ray diffraction wavelength (*λ* = 0.15406 nm), and *θ* is the diffraction angle.

The following Formula (2) can be used to calculate the microscopic strain (*ε*) [16,31]:

(2) $$\varepsilon =\frac{\beta cos\theta }{4}$$

where *θ* is the diffraction angle and *β* is the full width at half-maximum (FWHM).

In addition, the screw dislocation density of AlN can be evaluated by the following equation [16,31]:

(3) $$\delta =\frac{\beta_{\left(0002\right)}^{2}}{4.35b^{2}}$$

where *β* is the FWHM and *b* is the length of the Burgers vector, which is 0.3110 nm.

Through the above calculation, the crystallite size, micro-strain, and screw dislocation density values of two samples are obtained, as shown in Table 1. After comparison, the FWHM of AlNn is smaller than that of AlNt. The XRD symmetric (0002) linewidth was measured to be around 351 arcsec for AlNn and 437 arcsec for AlNt, respectively, indicating that the crystalline perfection of AlNn is better. Both AlN samples indicate low threading dislocation density. The thicker AlNn exhibits lower dislocations than another one; it is further proven that thick AlN epitaxial film can serve as a strain relief layer while significantly bending the dislocation and reducing total dislocation density. The reason can be explained below. With the increase of AlN thickness, the dislocation induced by the lattice mismatch between AlN epilayers and sapphire will gradually decrease and may even be annihilated, thus improving the crystal quality.

### 3.2. Optical Transmission Spectroscopy Analysis for AlN

**Figure 3:**
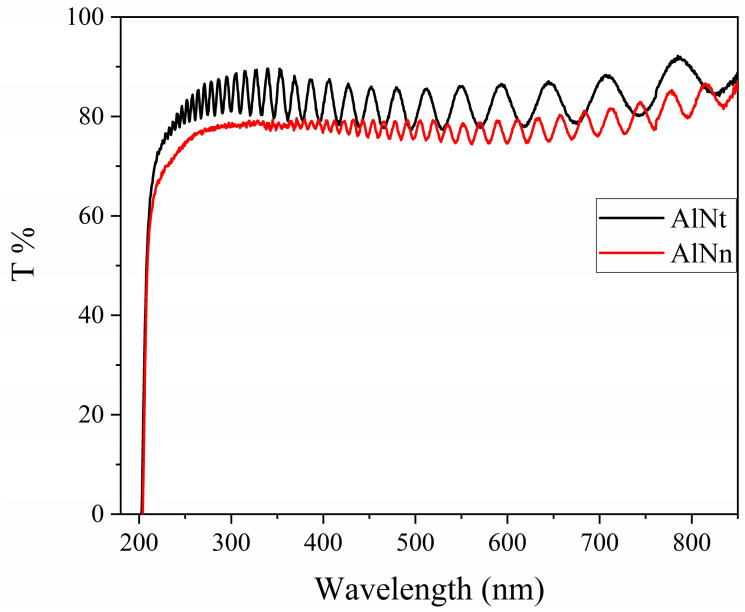
Optical transmission spectra of AlNt and AlNn.

Figure 3 shows the optical transmission (OT) spectra of AlNt and AlNn samples, measured by a dual-beam ultraviolet-visible spectrophotometer. It can be observed that the absorption cut-off lines of both samples are very steep, indicating the advantage of thick film AlN. In the meantime, the spectra of the two samples show clear oscillations below the absorption edge, that is, the transparent region beyond 300 nm, indicating the advantages of this method of sample preparation, which can grow a film with a uniform distribution texture and excellent quality.

To better compare the two samples, AlNt and AlNn, the dependence of the absorption coefficient on photon energy can be used through the formula [32]:

(4) $$\alpha hv=C{\left(hv-E_{g}\right)}^{1/2}$$

where *C* is a constant, $E_{g}$ is the bandgap of the semiconductor, and hv is the photon energy. 

**Figure 4:**
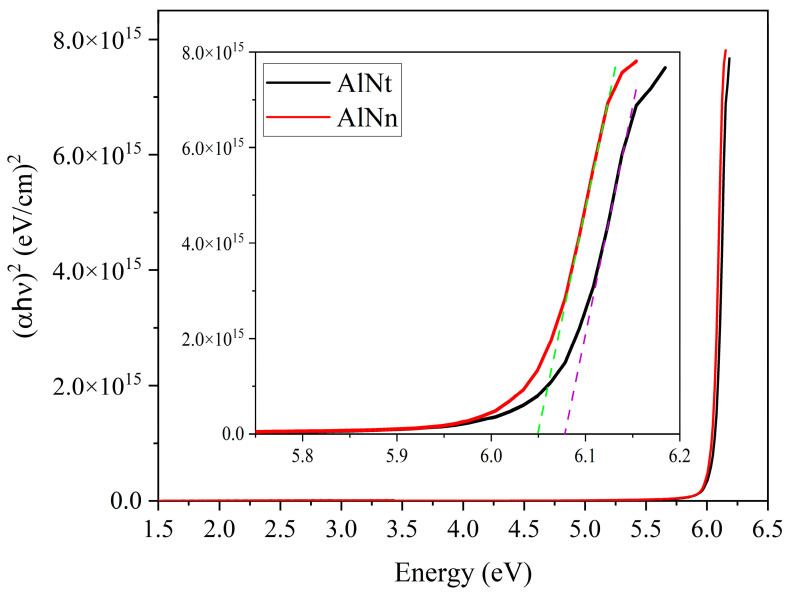
Calculated (αhv)^2^ vs. photon energy (hv) for two AlN epi-films by OT.

The optical bandgap energy (*E_g_*) of AlN can be obtained by extrapolating the linear part of the proportional dependence of (*α*hv)^2^ vs. photon energy (hv) by Formula (4), known as the Tauc plot [32]. The values of *E_g_* can be calculated by linear fit close to the absorption edge (Dotted lines in green and purple), as shown in smaller image of Figure 4. Where the smaller built-in image in Figure 4 is the enlarged view of Figure 4. Thus, the bandgap of the two samples can be seen in Figure 4: *E_g_* (AlNt) = 6.08 ± 0.03 eV and *E_g_* (AlNn) = 6.05 ± 0.03 eV, as shown in Table 2. 

**Figure 5:**
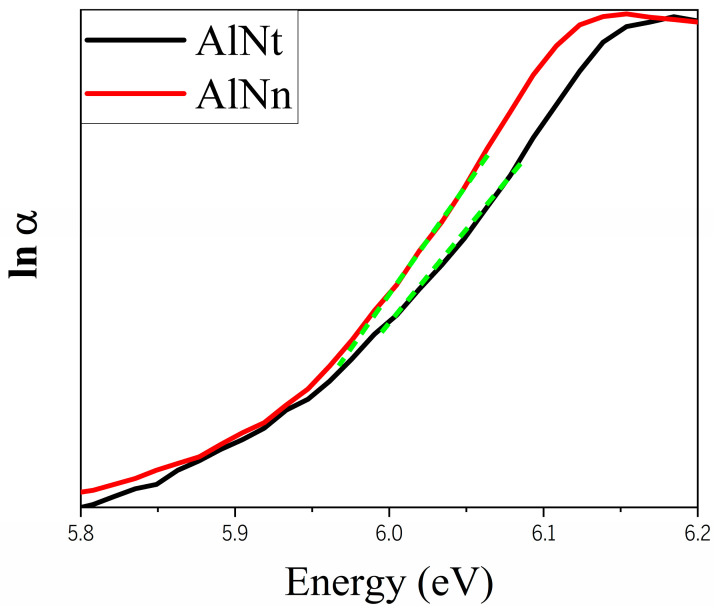
The relation between lnα and photon energy (hv) by OT.

Figure 5 exhibits exponential absorption band tails below the band edge for both AlN samples, which may result from structural disorder accompanying electron–phonon coupling [33,34]. The Urbach’s binding energy (E_u_), which is a band tail parameter, can be determined from the formula [34]:

(5) 1/Eu=d(lnα)/d(hv)

**Figure 6:**
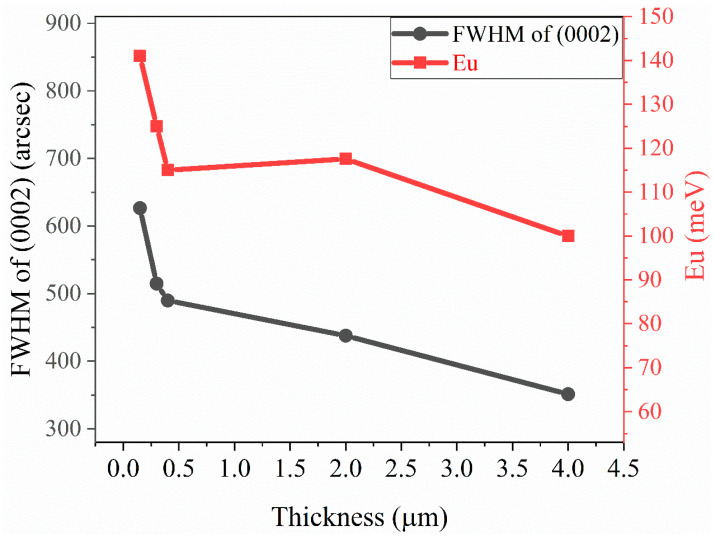
Comparison between FWHM (0002) by HR-XRD and Urbach’s binding energy (E_u_) as a function of AlN epi-film thickness by OT. The data for S1 (150 nm), S2 (300 nm), and S3 (400 nm) are from Ref. [17] for comparison.

Figure 6 shows Urbach’s binding energy E_u_ compared with the FWHM of (0002) vs. the thickness of AlN. We obtain the E_u_ of AlNt and AlNn by OT as 117.56 meV and 99.97 meV, respectively. It shows that the obtained E_u_ (red solid squares in Figure 6) is decreased with AlN epilayer thickness, which corresponds to the FWHM of HR-XRD results discussed before (black solid circles in Figure 6), i.e., an improvement of structural quality. The high correlation between OT-derived E_u_ and HR-XRD FWHM predicates that the AlN crystalline quality can be demonstrated from the spectral properties in the vicinity of the band edge. The data for S1 (150 nm), S2 (300 nm), and S3 (400 nm) are from Ref. [17] for comparison.

### 3.3. Cross-Sectional Morphologies of AlN

**Figure 7:**
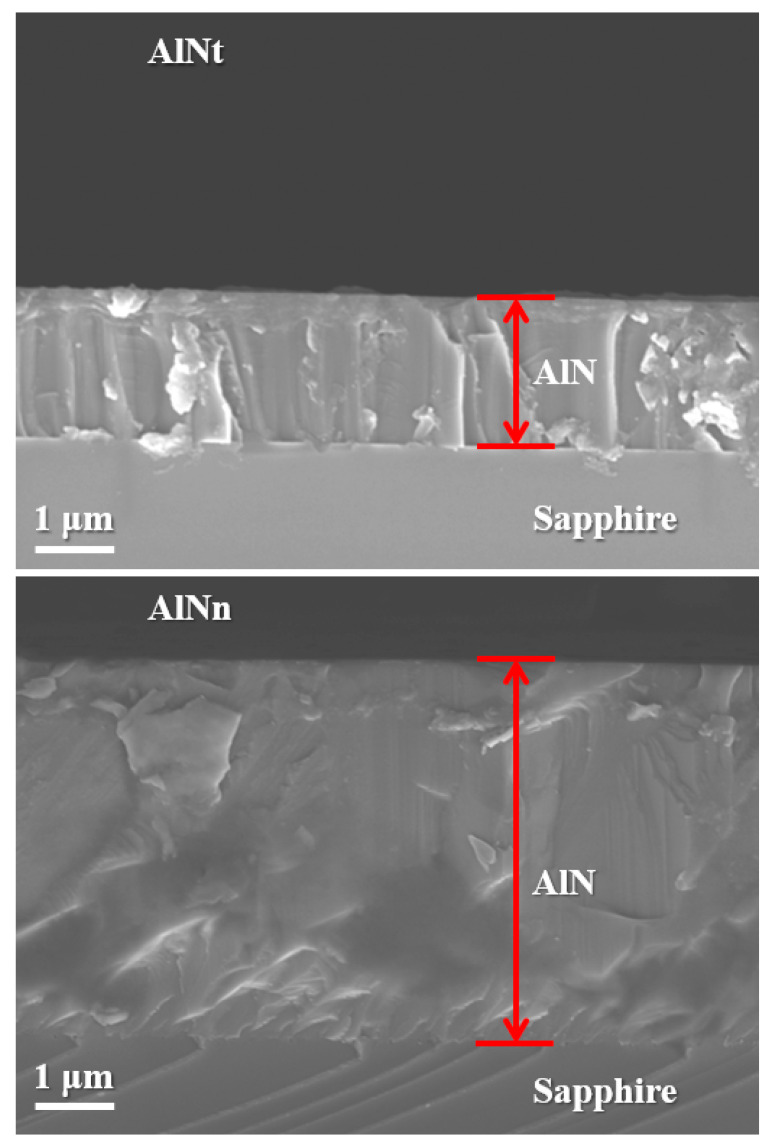
Cross-sectional SEM micrographs of AlNt and AlNn.

The thickness of the AlN film can also be determined by SEM on the AlN film cross-section. From Figure 7, it can be obtained that the thicknesses of AlNt and AlNn samples are about 1.93 μm (AlNt) and 4.29 μm (AlNn), respectively. It can be seen that the AlN epitaxial layer in sample AlNn gradually becomes tightly packed, indicating that the crystal density of the AlN epitaxial layer is higher for a thick film, which corresponds to the XRD characterization.

### 3.4. Spectroscopic Ellipsometry Analysis of AlN

Spectroscopic ellipsometry (SE) measurements were performed for two AlN/sapphire samples, showing the changes in polarization states psi (Ψ) and delta (Δ) between the incidence and reflection of light on the sample. SE spectra of AlN samples are fitted using the J.A. Woollam Co. software to establish a four-phase physical model, including roughness/epitaxial AlN layer/AlN buffer layer/sapphire substrate. After fitting, the thickness, optical constants, surface roughness, and bandgap energy of the AlN epitaxial layer were derived. In the fitting model, a Bruggeman effective medium approximation was used to model the surface roughness. The optical constants of a sapphire substrate from Ref. [35] were adopted and kept fixed in the fitting procedure. Two AlN layers are composed of a Gaussian oscillator, a PSemi-Tri oscillator, and a PSemi-MO oscillator. All parameters in AlN layers were adjusted to acquire the best-fitting SE data for two samples at room temperature (RT). The thicknesses of the AlN film, buffer layer, and surface roughness can be obtained within reasonable boundaries.

**Figure 8:**
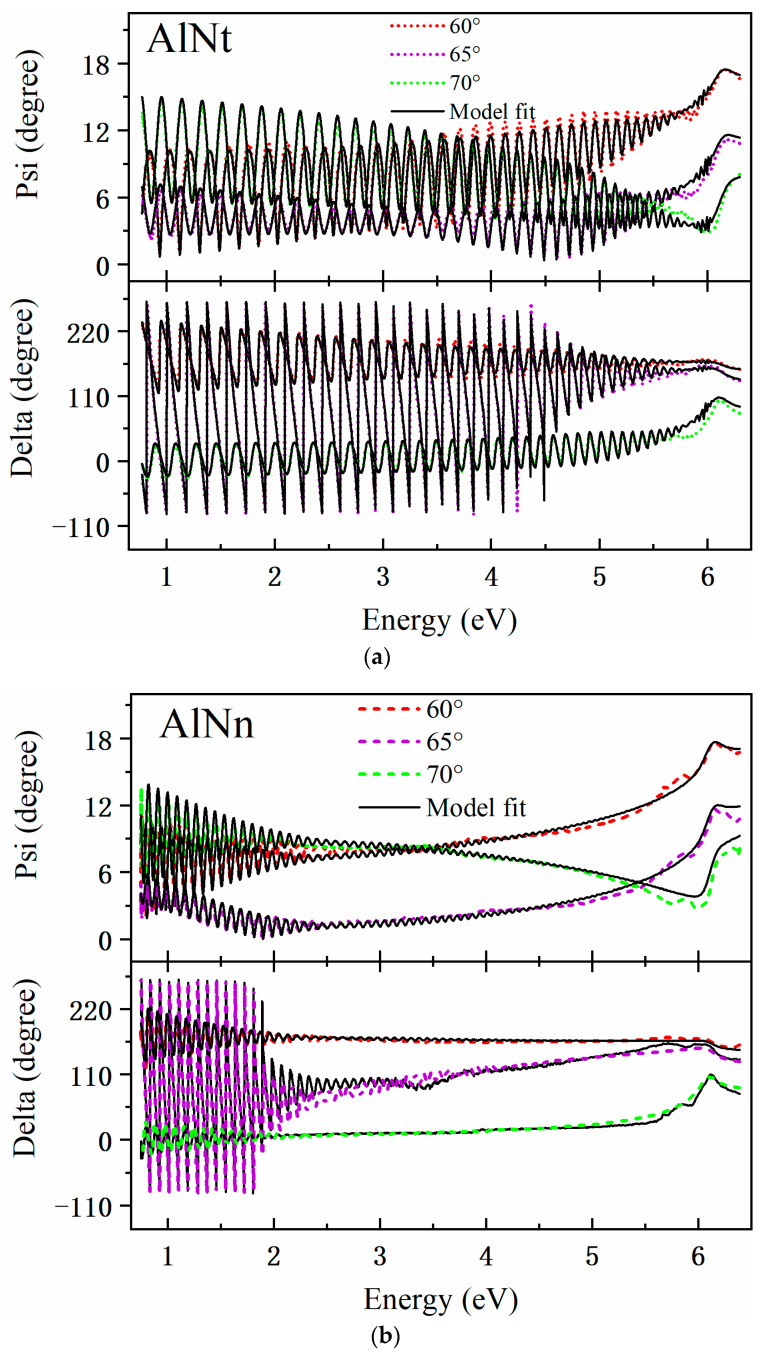
(**a**) Polarization states Ψ (*λ*) and Δ (*λ*) spectra of AlNt at room temperature, where the color dotted line and black solid line represent experimental measured data and model fitted data, respectively. (**b**) Polarization states Ψ (*λ*) and Δ (*λ*) spectra of AlNn at room temperature, where the color dotted line and black solid line represent experimental measured data and model fitted data, respectively.

**Figure 9:**
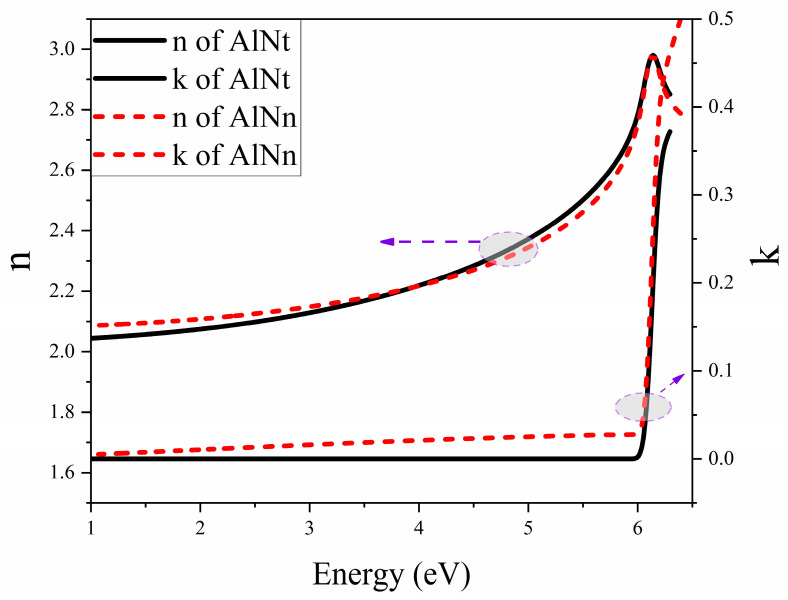
Fitted optical constants (refractive index n and extinction coefficient k) of two AlN samples at room temperature by SE.

**Figure 10:**
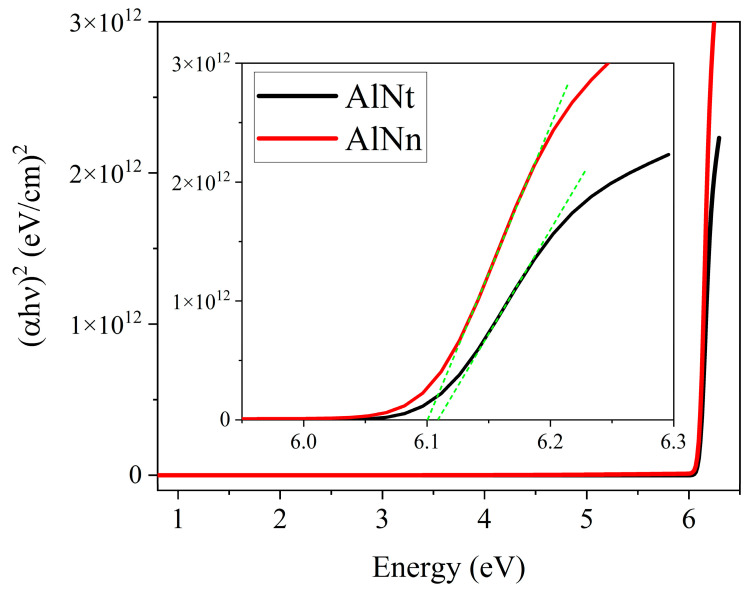
Calculated (αhv)^2^ vs. photon energy (hv) for two AlN epi-films by SE.

Figure 8a,b presents the experimental SE spectra and fitting curves of psi (Ψ) and delta (Δ) at 300 K with three incident angles of 60°, 65°, and 70° for two AlN samples. The experimental and fitting results are in good agreement. The interference oscillations below the bandgap edge correspond to the transparent region of the AlN sample. The final fitting results with parameters of the surface roughness, epilayer, and interlayer thicknesses for both AlN samples are listed in Table 2. In the meantime, the optical constants of AlNt and AlNn samples, such as refractive index (n) and extinction coefficient (k), vs. the photon energy, are extracted by SE fitting at 300 K and shown in Figure 9. The optical bandgap energy (*E_g_*) of AlN can be obtained by extrapolating the linear part of the proportional dependence of (*α*hv)^2^ vs. photon energy (hv) by Formula (4), known as the Tauc plot [32]. Thus, the bandgap of the two samples can be seen in Figure 10: *E_g_* (AlNt) = 6.11 ± 0.01 eV and *E_g_* (AlNn) = 6.10 ± 0.02 eV, as shown in Table 2.

**Figure 11:**
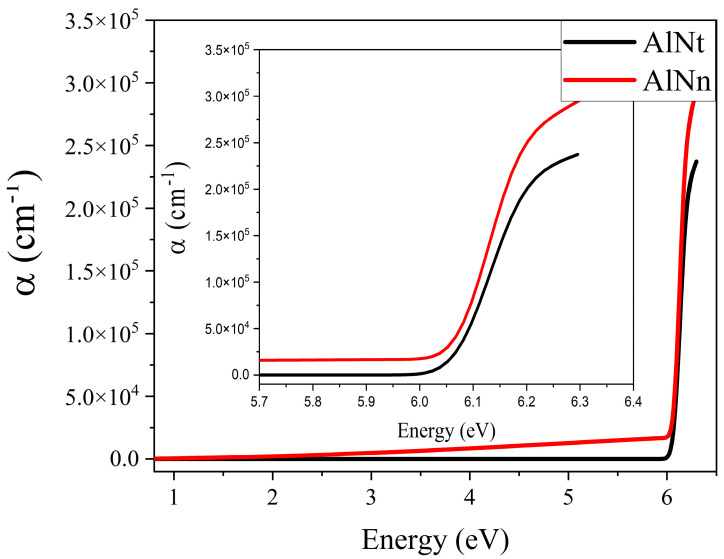
The absorption coefficient of two AlN samples at room temperature by SE.

**Figure 12:**
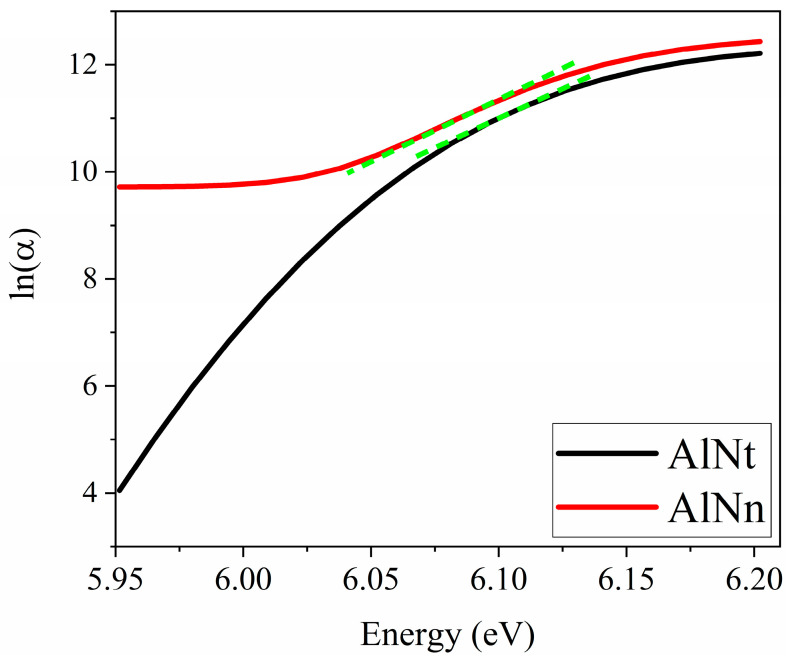
The relation between ln (α) and photon energy (hv) by SE.

**Figure 13:**
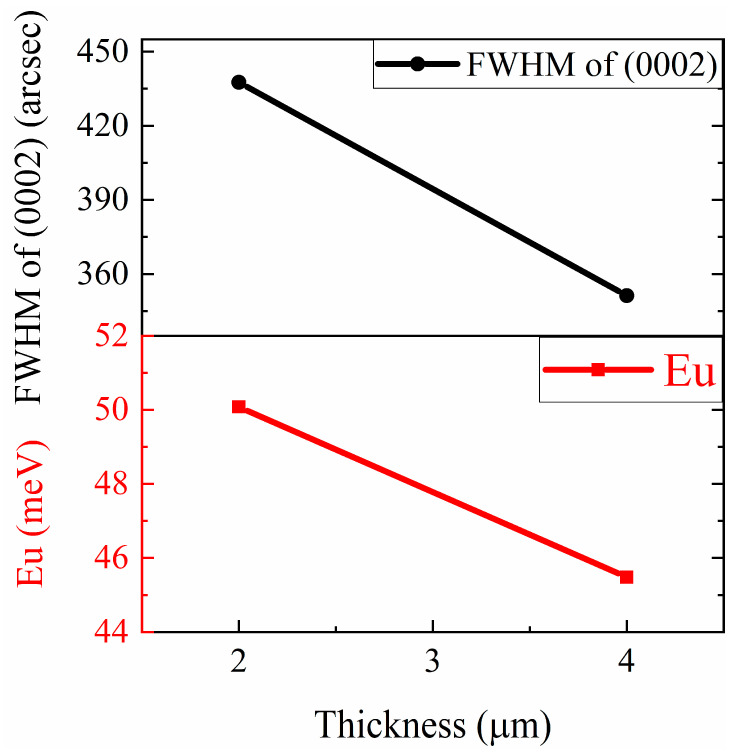
Comparison between FWHM (0002) by HR-XRD and Urbach’s binding energy (E_u_) as a function of AlN epi-film thickness by SE.

Figure 11 shows the absorption coefficient (*α*) of epilayer vs. photon energy (hv), according to *α* = 4πk/*λ*, where k is the extinction coefficient. From Figure 11, below the band-edge, the absorption band tail is observed for two AlN samples, which could originate from the structural disorder accompanying electron–phonon coupling [17]. The band tail parameters E_u_ of AlNt and AlNn can be obtained by Formula (5), which are 50.08 meV and 45.48 meV, respectively, as shown in Figure 12 and Table 2. Through comparison, it can be found that the changing trend of these two parameters from OT and SE is consistent with the results of XRD from Figure 13, which proves the accuracy of SE characterization. Meanwhile, it is also observed that the result of E_u_ obtained by SE is much smaller than OT. The reason is that the data from the OT test is the combination of the entire AlN epitaxial layer and buffer layer, while the data taken from SE is directly from the epitaxial layer. The epitaxial layer usually has better quality and a lower density of defects than the interlayer, so the E_u_ value is smaller by SE than by OT. The bandgap of two samples can be acquired by Formula (4) as shown in Figure 10: *E_g_* (AlNt) = 6.11 ± 0.01 eV and *E_g_* (AlNn) = 6.10 ± 0.02 eV, as indicated in Table 2. The values of the bandgap obtained by SE are slightly larger than those measured by OT. The reason is that the bandgap obtained by the OT method is the bandgap including interlayer and epi-layer, while the bandgap obtained by the SE method is only the bandgap of epi-layer, and the bandgap is affected by the band edge, so the Urbach band tail characterized by SE is smaller, resulting in a larger bandgap characterized by SE. With the increase in epitaxial layer thickness, some of the dislocations will annihilate, and the quality of AlN films will tend to be better.

### 3.5. Temperature-Dependent SE Analysis of AlN

**Figure 14:**
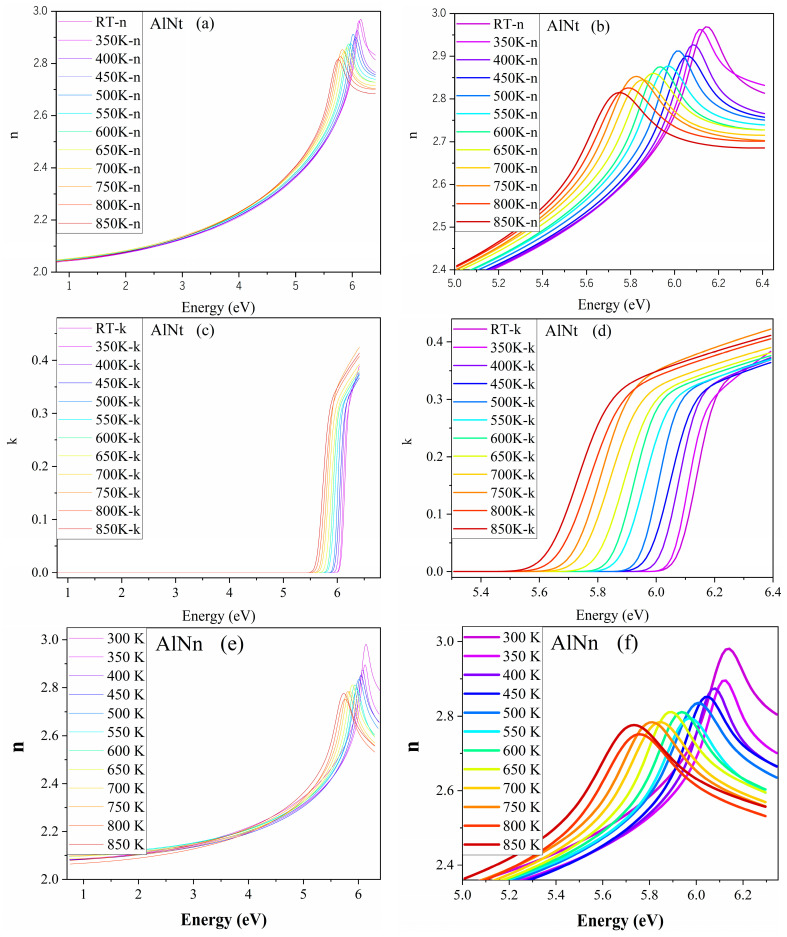

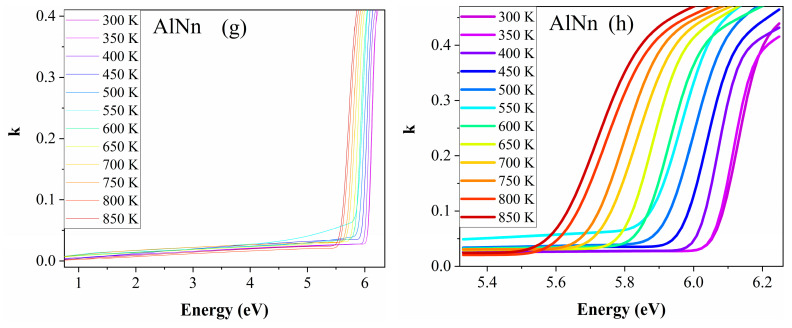
(**a**–**h**) n and k of AlNt and AlNn by VT-SE, where (**b**,**d**,**f**,**h**) are enlarged views of (**a**,**c**,**e**,**g**), respectively.

To understand the influence of temperature on the optical properties of AlN epi-films, a variable-temperature ellipsometry experiment was carried out. Based upon the accuracy of the SE dispersion model and fitting results, the room-temperature fitting parameters of the model were used as the initial values for subsequent high-temperature fitting. Then, the relevant parameters are adjusted, and the surface roughness, film, and interlayer thicknesses are further fitted to match the SE data of the high-temperature experiment. A special concern is the study of the changes in bandgap energy (*E_g_*), refractive index (n), and extinction coefficient (k) with temperature. Figure 14a–h show the refractive index (n) and extinction coefficient (k) vs. photon energy at different temperatures, where Figure 14b,d,f,h are enlarged views of Figure 14a,c,e,g, respectively. It indicates that the n and k values shift from right to left as the temperature increases.

**Figure 15:**
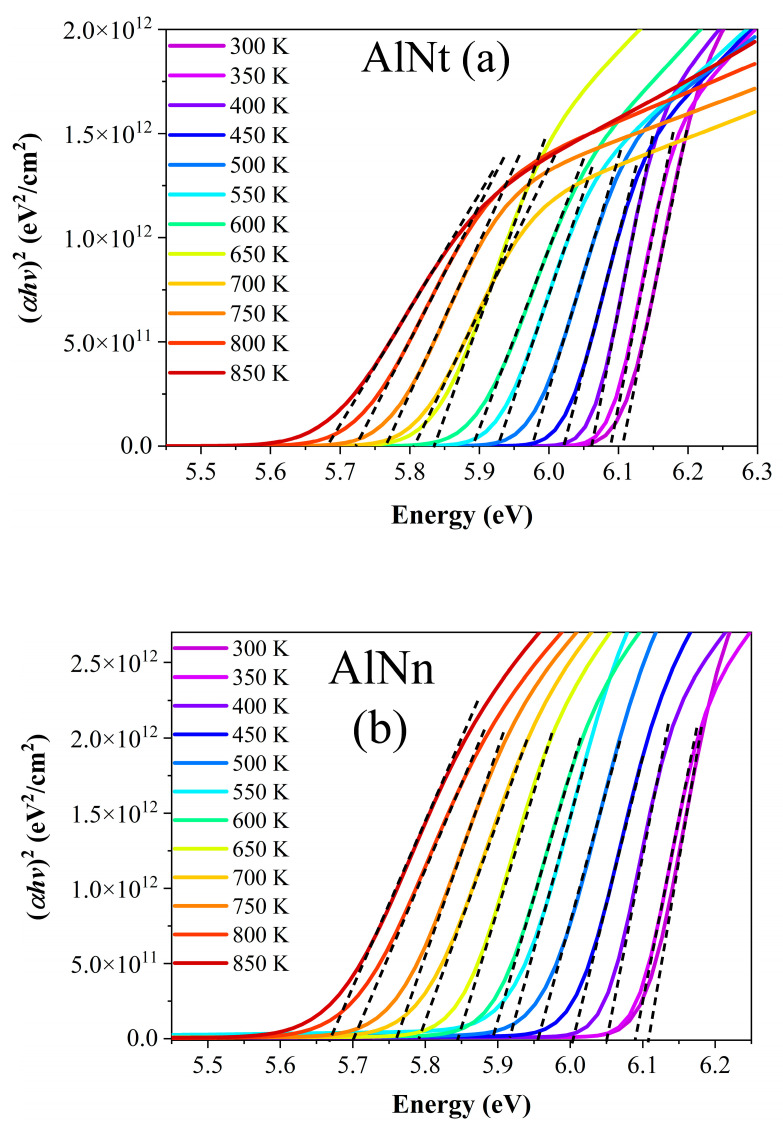
Relationship of (αhv)^2^ vs. photon energy (hv) for AlNt (**a**) and AlNn (**b**) epi-films by SE at a temperature range of 300–850 K.

**Figure 16:**
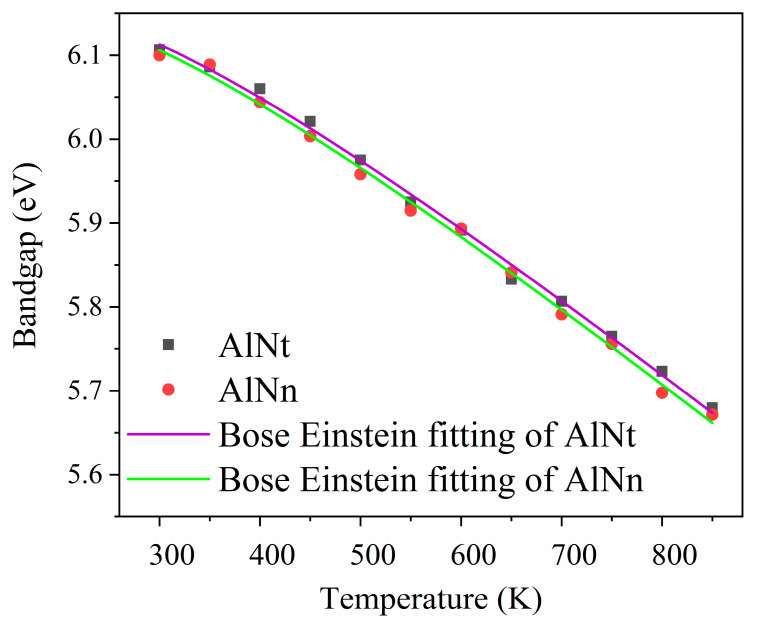
Temperature dependence of the bandgap energies from 300 K to 850 K for two AlN samples.

Figure 15a,b show (αhv)^2^ vs. photon energy (hv) of AlNt and AlNn at 12 temperature points between 300 K and 850 K. The optical bandgap energy (*E_g_*) of AlN can be obtained by extrapolating the linear part of the proportional dependence of (*α*hv)^2^ vs. photon energy (hv) by Formula (4) [32]. It is observed that with the temperature rising, the absorption edge shifts to the lower energy, together with the enlarged band tailing, i.e., the redshift of the bandgap. Figure 16 depicts the dependence of the bandgap on temperature (*T*) for the two AlN films. The bandgap energy of both samples exhibits an apparent redshift with the increase in temperature. These results are due to the dominant electron–phonon interactions being stronger than the weak contribution of thermal expansion [36,37].

The change of the bandgap with temperature can be fitted using Bose–Einstein’s analytical formula [29],

(6) $$E_{g}\left(T\right)=E_{g}\left(T=0\;K\right)-2\alpha_{B}/[\exp\left(\frac{\theta }{T}\right)-1]$$

where *θ* is related to the average phonon temperature,$\;\alpha_{B}$ represents the strength of the average electron–phonon coupling. The fitting results are presented in Figure 16 and Table 3. It is seen that the fitting degree of accuracy is very high due to one of the values being extremely close to a value of 1 for Adj.R-Square. The solid lines in Figure 16 show the trend of the bandgap with temperature. Table 3 also lists the fitted values of $\alpha_{B}$ and *θ* from Ref. [17] for comparison. We can see that the reported data in Ref. [17] are all larger than those obtained by our present work, indicating that the larger the thickness, the weaker the electron–phonon coupling and the following slower decline of *E_g_*. However, in this study, the $\alpha_{B}$ of the two samples was similar, and the declining value of *E_g_* was similar. This result may predict that when the thickness increases to a certain value (micrometer order), the thickness has little effect on the change of *E_g_* with temperature, which indicates the thermal stability of *E_g_* for thick AlN films. Combined with the changing trend of the Urbach band tail of five samples in Figure 6, we can also find that the declining trend of the Urbach band tail E_u_ becomes smaller when the thickness of the sample reaches the micron level. The reason for these two kinds of similar phenomena may be that when the thickness reaches the micron level, all the islands in the growth of AlN coalesce together, and the dislocations induced by lattice mismatch diminish gradually, so the influence of thickness on thermal stability will become smaller.

## 4. Conclusions

In summary, the optical and structural characteristics of AlN films grown on sapphire substrates by MOCVD have been investigated by way of HRXRD, OT, SEM, and SE, especially focused on optical properties at high-temperatures by temperature-dependent SE. The thicker AlNn exhibits lower dislocations than the thinner ones; it is further proven that thick AlN epitaxial samples can serve as a strain relief layer, significantly bend the dislocation, and reduce total dislocation density. The reason is that, with the increase in AlN thickness, the dislocation arising from the lattice mismatch between AlN and sapphire will be gradually decreased and may even be annihilated, thus improving the sample’s crystalline quality. Urbach’s binding energy E_u_ and optical bandgap *E_g_* by OT and SE depend on crystallite alignment, crystalline size, and film thickness, which are highly by XRD and SEM results. With the combination of SE, HRXRD, and OT analyses, we concluded that with the treatment of thickening film, the essence of crystal quality improved. However, the bandgap energies obtained from SE have larger values and higher accuracy than those extracted from OT. It has been proven that SE measurement is an efficient and useful method to characterize semiconductor thin films. The Bose–Einstein relation is used to demonstrate the temperature dependence of the bandgap. Our results reveal that the thermal stability of the AlN bandgap energy can be improved by increasing film thickness, which is caused by the corresponding weaker electron–phonon interactions. However, when the thickness increases to micrometer order, the thickness has little effect on the change of *E_g_* with temperature. The AlN film with epilayer thicknesses of 1.776 and 3.666 µm has a small average electron–phonon coupling of about 390 meV. These results and analyses will provide good information for further penetrative research for AlN-based devices such as SAW sensors, LDs, LEDs, and other optoelectronic devices, especially those working at high temperatures.

## Figures and Tables

**Table 1:** (0002), (0004), and (0006) ω FWHM, calculation results of crystallite size, micro-strain, and dislocation density of two AlN epi-films.

		AlNt	AlNn
FWHM_(0002)ω_	(arcsec)	438 ± 4	351 ± 5
FWHM_(0004)ω_	(arcsec)	434 ± 5	355 ± 6
FWHM_(0006)ω_	(arcsec)	531 ± 8	456 ± 8
Crystallite size	(nm)	68.72 ± 0.02	85.62 ± 0.03
Micro-strain	(×10^−3^)	0.50 ± 0.04	0.41 ± 0.05
Screw dislocation density	(×10^−5^) (nm^−2^)	1.07 ± 0.05	0.69 ± 0.06

**Table 2:** A list of SE fitting results and OT results of AlN samples.

Sample Name	AlNt	AlNn
Surface roughness by SE (nm)	3.07 ± 0.03	3.58 ± 0.05
Thickness of epilayer by SE (nm)	1776.35 ± 0.04	3666.17 ± 0.05
Thickness of the buffer layer by SE (nm)	21.27 ± 0.06	51.89 ± 0.06
Thickness of AlN by SEM (μm)	1.93 ± 0.05	4.29 ± 0.05
Bandgap by SE (eV)	6.11 ± 0.01	6.10 ± 0.02
Bandgap by OT (eV)	6.08 ± 0.03	6.05 ± 0.03
E_u_ by SE (meV)	50.08 ± 0.02	45.48 ± 0.02
E_u_ by OT (meV)	117.56 ± 0.03	99.97 ± 0.04

**Table 3:** A list of fitting parameters for $\alpha_{B}$ and θ from AlN samples.

Sample Name	Thickness	$\alpha_{B}$	θ	Adj.R-Square	Reference
	(nm)	(meV)	(K)		
AlNt	1776.35	389 ± 100	800 ± 154	0.99645	This work
AlNn	3666.17	390 ± 102	795 ± 155	0.99639	This work
S1	136.42	687 ± 72	1233 ± 73		Ref. [17]
S2	307.85	554 ± 54	1111 ± 64		Ref. [17]
S3	412.90	407 ± 36	977 ± 58		Ref. [17]

## Data Availability

The data presented in this study are available on request from the corresponding authors.
